# PEG 400:Trehalose Coating Enhances Curcumin-Loaded PLGA Nanoparticle Internalization in Neuronal Cells

**DOI:** 10.3390/pharmaceutics15061594

**Published:** 2023-05-25

**Authors:** Isaac H. Caballero-Florán, Hernán Cortés, Fabiola V. Borbolla-Jiménez, Carla D. Florán-Hernández, María L. Del Prado-Audelo, Jonathan J. Magaña, Benjamín Florán, Gerardo Leyva-Gómez

**Affiliations:** 1 Departamento de Farmacología, Centro de Investigación y de Estudios Avanzados del Instituto Politécnico Nacional, Ciudad de México 07360, Mexico; icaballero@cinvestav.mx; 2 Departamento de Farmacia, Facultad de Química, Universidad Nacional Autónoma de México, Ciudad Universitaria, Circuito Exterior S/N, Del. Coyoacán, Ciudad de México 04510, Mexico; 3 Laboratorio de Medicina Genómica, Departamento de Genómica, Instituto Nacional de Rehabilitación-Luis Guillermo Ibarra Ibarra (INR-LGII), Ciudad de México 14389, Mexico; hcortes@inr.gob.mx (H.C.); fvj2312@gmail.com (F.V.B.-J.); jmagana@inr.gob.mx (J.J.M.); 4 Departamento de Fisiología, Biofísica & Neurociencias, Centro de Investigación y de Estudios Avanzados, del Instituto Politécnico Nacional, Ciudad de México 07360, Mexico; daniela.floran@fisio.cinvestav.mx; 5 Tecnologico de Monterrey, Escuela de Ingeniería y Ciencias, Campus Ciudad de México 14380, Mexico; luisa.delprado@tec.mx

**Keywords:** trehalose, polyethylene glycol, PEG, nanoprecipitation, nanoparticles, PLGA, neurons, cell internalization, drug delivery

## Abstract

This work proposes a combination of polyethylene glycol 400 (PEG) and trehalose as a surface modification approach to enhance PLGA-based nanoparticles as a drug carrier for neurons. PEG improves nanoparticles’ hydrophilicity, and trehalose enhances the nanoparticle’s cellular internalization by inducing a more auspicious microenvironment based on inhibiting cell surface receptor denaturation. To optimize the nanoprecipitation process, a central composite design was performed; nanoparticles were adsorbed with PEG and trehalose. PLGA nanoparticles with diameters smaller than 200 nm were produced, and the coating process did not considerably increase their size. Nanoparticles entrapped curcumin, and their release profile was determined. The nanoparticles presented a curcumin entrapment efficiency of over 40%, and coated nanoparticles reached 60% of curcumin release in two weeks. MTT tests and curcumin fluorescence, with confocal imaging, were used to assess nanoparticle cytotoxicity and cell internalization in SH-SY5Y cells. Free curcumin 80 µM depleted the cell survival to 13% at 72 h. Contrariwise, PEG:Trehalose-coated curcumin-loaded and non-loaded nanoparticles preserved cell survival at 76% and 79% under the same conditions, respectively. Cells incubated with 100 µM curcumin or curcumin nanoparticles for 1 h exhibited 13.4% and 14.84% of curcumin’s fluorescence, respectively. Moreover, cells exposed to 100 µM curcumin in PEG:Trehalose-coated nanoparticles for 1 h presented 28% fluorescence. In conclusion, PEG:Trehalose-adsorbed nanoparticles smaller than 200 nm exhibited suitable neural cytotoxicity and increased cell internalization proficiency.

## 1. Introduction

Interest in natural products with biological activity has led to the studying of natural extracts as emerging treatments for different disorders. Concerning this, curcumin is a natural polyphenol with antioxidant and anti-inflammatory activity that has demonstrated high potential against brain diseases [1,2,3,4]. Curcumin has unpaired electrons, which make this compound a potent antioxidant [4,5]. However, curcumin shows low availability after oral administration. Additionally, this compound presents chemical properties that render it a challenge to formulate, including high hydrophobicity and photosensitivity. Thus, strategies based on nanoparticles have been proposed to elaborate drug delivery systems for curcumin. These nanosystems might improve curcumin’s bioavailability and ability to cross biological barriers [6,7].

Effective cellular internalization directly impacts the nanoparticle’s capacity to carry drugs and produce a therapeutic effect [8,9]. Therefore, a crucial characteristic of the nanoparticles for medical purposes is the ability to internalize the target cell after entering a biological system. Fundamental nanoparticle core properties such as size, polydispersity index (Pdi), shape, stiffness, and particle surface chemistry improve cell–nanoparticle interaction. Nevertheless, biological characteristics, including cell morphology, cell type (tissue), the microenvironment, and cellular disease states, also impact the interaction–internalization cell process [10]. Therefore, properly selecting materials to make nanoparticles with suitable properties is a significant aspect of transporting drugs into the cells.

Among the nanoparticle-based drug delivery systems, polymer-based nanoparticles are considered reliable because they enhance drug solubility, reduce drug toxicity, and allow nanoparticle surface modification [8,11]. A remarkable example of synthetic polymers is poly(lactic-co-glycolic acid) (PLGA), which the Food and Drug Administration recognizes as a biomaterial with a desired level of biocompatibility and biodegradability [8,9,11]. The physical and chemical properties of PLGA are helpful in the optimization process for producing nanoparticles [12,13,14]. Moreover, PLGA nanoparticles can be modified in size and shape according to their intended use. Although PLGA’s carboxyl groups produce nanoparticles with a high zeta potential (−25 mV) that are extremely stable, the high surface charge may complicate their interaction with cellular membranes and cause a reduction in cell internalization [11,15]. In this context, surface modification strategies may enhance the optimal uptake of nanoparticles by cells.

Several molecules may be used to change the surface of nanoparticles, although polyethylene glycol (PEG) is the most frequently used. PEG is a member of molecules known as shielding groups and may be grafted or adsorbed onto the surfaces of nanoparticles [16,17,18]. PEG reduces opsonization and complement system activation, protecting nanoparticles from the mononuclear phagocytic system. PEG also possesses a strong hydrophilicity, a large spatial repulsion, and a neutral electric charge. Due to these characteristics, the nanoparticles’ circulation half-life is increased several times [17,19]. In this regard, the molecular weight (MW) of the PEG used to modify nanoparticle surfaces has demonstrated a high influence on the interaction of the nanoparticles with the biological fluids and cell membranes. For example, PEG-coated nanoparticles improved cell internalization when PEG MW as well as coverage density increased [17,20]. Likewise, PEG brush conformation easily alters membranes when the PEG MW is higher, which increases the internalization time [21,22]. Contrariwise, a low PEG MW and a high PEG coverage in the nanoparticles result in rapid mucus penetration [12,13,14].

Likewise, trehalose has also gained importance as a chemical that aids in preserving biological molecules and cells under oxidative and environmental stress. Trehalose is a natural disaccharide present in microorganisms and plants. The trehalose molecule contains non-reducing end hydroxyl (−OH) groups, making it a stable molecule unable to participate in glycation reactions or prevent their metabolism. Molecules of trehalose can be detected in peripheral circulation 30 min after oral administration in rodents [23]. Trehalose is also reported in various studies to suppress protein aggregation in the intracellular/extracellular space and to destroy mature protein fibrils [24]. Trehalose can also provide a microenvironment that makes it easier for cells to absorb nanoparticles, providing an unfunctionalized cellular internalization route. This process is linked to the ability of trehalose to mediate the inhibition of cell surface receptor denaturation, which is one of the primary causes of membrane lysis and cell death caused by nanoparticles [25].

Here, we propose a PEG and trehalose coating technique to modify the surface of the PLGA nanoparticles loaded with curcumin. We tested to determine if the modification affected the nanoparticle’s performance as a drug carrier, as well as its ability to internalize in neural cells.

## 2. Materials and Methods

### 2.1. Materials

Poly(D,L-lactide-co-glycolide) 50:50 Mw 24,000-38, (Resomer^®^ RG 503 H), Poly(vinyl alcohol) (Mowiol^®^), (E,E)-1,7-bis(4-Hydroxy-3-methoxyphenyl)-1,6-heptadiene-3,5-dione (Curcumin, from *Curcuma longa* (Turmeric), powder), α-D-Glucopyranosyl-α-D-glucopyranoside (Trehalose dihydrate), Poly(ethylene glycol) (Kollisolv^®^ PEG E 400), and Dodecyl sodium sulfate (Sodium Laureth sulfate) were purchased to Sigma-Aldrich Merck (St. Louis, MO, USA).

### 2.2. Preparation and Optimization of PLGA Nanoparticles

#### 2.2.1. Nanoprecipitation Technique

We synthesized PLGA blank nanoparticles (BNPs) using the nanoprecipitation method [26]. First, we prepared two solutions: an aqueous solution of polyvinyl alcohol (PVA; aqueous phase) and another one with PLGA dissolved in acetone (organic phase). We maintained the aqueous phase at room temperature under continuous magnetic agitation while the organic phase was added dropwise at 0.333 mL/min. Then, we removed acetone through constant agitation for 12 h at 200 rpm, at room temperature. The next day, we resuspended nanoparticles in distilled water for the subsequent analysis.

#### 2.2.2. Experimental Design

We optimized BNPs synthesis using a central composite design (CCD), an augmented factorial design, and a preset with StatGraphics Centurion XVI.II Software (The Plains, VA, USA). We settled CCD parameters to estimate response surfaces (RSM) and optimize the response variables (Size (*y*_1_) and Pdi (*y*_2_)). We selected the PLGA amount (*x*_1_), PVA concentration [% *w*/*v*] (*x*_2_), and stirring speed (*x*_3_) as independent variables (critical factors in nanoparticle synthesis). For the factorial 2^3^ design, we chose low (−1) and high (+1) levels for each factor. To estimate the experimental error, the center point coded as 0 was set with six timed runs. Six more runs, called axial (star) points and coded as ±α, were inserted into the factorial 2^3^ design to construct the CCD. Axial points to the center point were set to configure a rotatable design and were described as α = 2k/4 (k is the number of independent variables); for this design, ±α = 1.68. The CCD 2^3^ + star design studied the effects of the three factors in 60 experiments [13,27]; we transcribed CCD-codified values in Table 1. The design was processed in three blocks. The order of the experiments was fully randomized to protect against the effects of lurking variables.

### 2.3. Curcumin Loading in Nanoparticles

After the nanoprecipitation technique optimization was finished, we used the best conditions to obtain nanoparticles and entrap curcumin. We added 1, 1.6, 2, 3, or 4 mg of curcumin to the organic phase to fabricate distinct nanoparticle batches with curcumin entrapped. We quantified free curcumin using spectrophotometry from the supernatants of the purification procedure. Curcumin amounts were determined using spectrophotometry at λ = 419 nm in a previously validated method (UV–visible spectrophotometer SP-V1000 DLAB). We calculated entrapment efficiency (%EE) using Equation (1). Once the curcumin-loaded nanoparticles (CNPs) were purified, we characterized their size, zeta potential, and Pdi.

(1) $$\%EE\;=\;\frac{\left(Amount\;of\;curcumin\;initial\;-\;Amount\;of\;curcumin\;free\right)}{\left(Amountofcurcumininitial\right)}\times 100$$

### 2.4. Nanoparticles PEG:Trehalose Coating

We performed the surface modification of BNPs and CNPs using the classic adsorption method, adjusted from the procedure settled by Ossama et al. [17,28]. BNPs or CNPs were resuspended in solutions with increasing equal concentrations of PEG and trehalose (2.5, 6.5, 8.0, and 12.5% *v/v* and *w*/*v*), respectively, for 12, 24, and 48 h, at room temperature. To concentrate these solutions, particles were centrifuged at 12,500 rpm for 20 min after the incubation period was complete. After reconstituting the nanoparticles in water, we measured the size, Pdi, and zeta potential. Furthermore, BNPs and CNPs were frozen at −20 °C for 24 h to evaluate the successful nanoparticle surface coating. Subsequently, nanoparticles were lyophilized at −60 °C and 2 Pa for 48 h (Scientz-10N Freeze Dryer, Ningbo, China). After the lyophilization process, the nanoparticles were stored in a desiccator to further characterization.

### 2.5. Nanoparticles Characterization

#### 2.5.1. Size, Polydispersity Index (Pdi), and Zeta Potential Measurements

We measured nanoparticle size and Pdi using the dynamic light scattering technique (DLS), while zeta potential was measured using the electrophoretic light scattering technique. We performed both measurements in the equipment Zetasizer Nano ZS 90 (Malvern instruments, Malvern, UK). The graphs, calculations, and statistical analyses were performed using GraphPad Prism Software version 8.0 (GraphPad Software, San Diego, CA, USA).

#### 2.5.2. Fourier-Transform Infrared Spectroscopy (FTIR)

Lyophilized samples of BNPs, CNPs, BNPs coated with PEG and trehalose (BNPs-PT), CNPs covered by PEG and trehalose (CNPs-PT), and raw materials were analyzed in a Spectrum Two IR spectrometer (Perkin Elmer, Waltham, MA, USA). The graphs were performed using Spectragryph version 1.2.15 (Software for optical spectroscopy, Oberstdorf, Germany).

#### 2.5.3. Differential Scanning Calorimetry (DSC)

For evaluating the thermal properties of BNPs, CNPs, BNPs-PT, CNPs-PT, and raw material, lyophilized samples were tested in hermetic aluminum cells using a DSC 2910 (Modulated TA Instruments^®^, New Castle, DE, USA). Measurements were performed from room temperature to 250 °C, at a heating rate of 10 °C/min, and under a nitrogen atmosphere. The graphs were performed using Spectragryph version 1.2.15 (Software for optical spectroscopy, Oberstdorf, Germany).

#### 2.5.4. Thermogravimetric Analysis (TGA)

We tested the thermal stability of BNPs, CNPs, BNPs-PT, CNPs-PT, and raw materials. The lyophilized samples of about 5 mg were placed in a Hi-Res TGA 2950 Thermogravimeter Analyzer (TA Instruments^®^, USA). Tests were carried out from room temperature to 500 °C, at a heating rate of 10 °C/min, and under a nitrogen atmosphere. The graphs were performed using Spectragryph version 1.2.15 (Software for optical spectroscopy, Oberstdorf, Germany).

### 2.6. In Vitro Release Study of Curcumin

We used freshly synthesized CNPs and CNPs-PT in the curcumin release study. For each formulation, we placed five independent batches in individual dialysis bags (MWCO 12–14 kDa) and immersed them in PBS [0.01M] pH 7.4, 2.5% (*w*/*v*) Sodium lauryl sulfate as dissolution media. We collocated the dissolution systems inside a water bath over a 10-position digital magnetic hotplate stirrer (IKA RT 10, Wilmington, NC, USA) with continuous stirring at 100 rpm. We kept the bath temperature at 38.5 °C with a compact immersion circulator (IKA ICC basic Wilmington, NC, USA) and took samples of 2 mL at predetermined time points for two weeks. We replaced the sample volume withdrawn with the same volume of fresh medium. We added 1 mL of acetone to each sample, and we calculated the molar adsorption coefficient (ε_419nm_ = 0.2697 mM^−1^ × cm^−1^) of released curcumin from the nanoparticles. Each aliquot was measured and plotted vs. the time, and both dissolution efficiency (DE) and mean dissolution time (MDT) were calculated. The graphs, calculations, and statistical analyses were performed using GraphPad Prism software version 8.0 (GraphPad Software, San Diego, CA, USA) Additionally, we evaluated the goodness of fit in different kinetic release models for both formulations with DDSolver Excel complement [29].

### 2.7. In Vitro Cytotoxicity Assay and Cellular Uptake

#### 2.7.1. Cell Culture

We assessed the cytotoxic effects of curcumin, BNPs, CNPs, BNPs-PT, and CNPs-PT in the neuroblastoma cell line SH-SY5Y. SH-SY5Y which are human neuroblastoma cells obtained from ATCC^®^ CRL-2266TM (Manassas, VA, USA). Cells were grown in DMEM-F12: MEM 1:1 medium supplemented with 10% FBS and 1% penicillin/streptomycin at 37 °C and 5% CO_2_ under a fully humidified atmosphere [30,31]. The cell culture medium was changed every 48 h.

#### 2.7.2. In Vitro Cytotoxicity Assay

After nanoparticle treatment, we determined the SH-SY5Y cell line viability with an MTT assay. First, cells were seeded in a 96-well plate 24 h before the assay. The cells were then incubated with curcumin, CNPs, CNPs-PT (2, 5, 10, 20, 40, and 80 μM), or corresponding samples of BNPs and BNPs-PT at 24 h (30,000 cells/well), 48 h (15,000 cells/well), or 72 h (8000 cells/well) [32,33,34]. After treatment, we added 8 μL of MTT solution 10 mg/mL in 92 μL of fresh growth medium per well, and cells were incubated at 37 °C for 4 h. Then, we added a solution of SDS 10% in HCl 0.1 M to solubilize the formazan crystals. Finally, plates were analyzed in a Synergy HTX multi-mode microplate reader (BioTek^®^, Winooski, VT, USA) at 590 nm. A negative control (growth medium) and positive control (10% ethanol) were included. All measurements were conducted in three independent experiments for triplicate.The graphs, calculations, and statistical analyses were performed using GraphPad Prism Software version 8.0 (GraphPad Software, San Diego, CA, USA).

#### 2.7.3. In Vitro Nanoparticles Cell Uptake

First, we seeded 10,000 SH-SY5Y cells on each coverslip for 24 h. Then we treated the cells with 100 μM curcumin, CNPs, CNPs-PT, or corresponding samples of BNPs and BNPs-PT for 1 and 4 h at 37 °C and 5% CO_2_. After incubation, we washed each coverslip two times with PBS 1X, fixed with paraformaldehyde 4% in PBS 1X for 20 min, and three times with PBS 1X. Coverslips were air-dried, mounted on a slide with 8 μL of mounting medium with DAPI, and sealed. We added a negative control incubated without treatment. All the process was carried out in the darkness. Cells were analyzed using a laser confocal scanning microscope (Leica TCS-SPE9 equipped with a 40× oil-immersion objective). We used Leica LAS AF lite software (Leica Microsystems, Wetzlar, Germany) to acquire the images, and ImageJ software was used for image analysis (Available online: imagej.nih.gov/ij (accessed on 13 October 2022)). The graphs, calculations, and statistical analyses were performed using GraphPad Prism Software version 8.0 (GraphPad Software, San Diego, CA, USA).

## 3. Results and Discussion

### 3.1. Optimization of Nanoprecipitation Technique for BNPs

We performed nanoprecipitation optimization experiments, and the results are summarized in Table 2. We measured the size and Pdi as effects of the independent variables: PLGA amount (*x*_1_), stirring speed (*x*_3_), and PVA concentration (*x*_2_). Particle size (*y*_1_) mean value ranged from 235.18 nm to 174.33 nm, and Pdi (*y*_2_) varied from 0.120 to 0.2498 (Table 2). From experimental data, the adjusted model Equations (2) and (3) were obtained for particle size and Pdi, respectively.

(2) $$y_{1}=205.814+14.3768x_{1}+2.03629x_{2}-10.8308x_{3}-1.03532x_{1}^{2}+1.065x_{1}x_{2}-1.7625x_{1}x_{2}+0.449018x_{2}^{2}-3.62083x_{2}x_{3}-2.41183x_{3}^{2}$$

(3) $$y_{2}=0.160372-0.0243843x_{1}-0.000299583x_{2}+0.0116046x_{3}+0.0169607x_{1}^{2}-0.00116667x_{1}x_{2}+0.0231667x_{1}x_{2}+0.00134541x_{2}^{2}-0.0145x_{2}x_{3}-0.00853436x_{3}^{2}$$

The size analysis of variance (Table 3) showed significant influence from two process variables: PLGA amount and stirring speed. We performed the lack of fit test to determine if the model adequately described the observed data, comparing the variability of the current model residuals to the variability between observations at replicated settings of the factors. Table 3 shows a *p*-value for lack of fit greater than 0.05, which means the model is adequate for 95% of the observed data.

In the case of Pdi analysis of variance (Table 4), this exhibited significant influence from five process variables: PLGA amount (*x*_1_), stirring speed (*x*_3_), the correlation between them (×1: ×3), autocorrelation (*x*_1_: *x*_1_), and the correlation between PVA concentration (*x*_2_) with stirring speed (*x*_3_) at 95%. The *p*-value for lack of fit was higher than 0.05, which means the model is adequate for 95% of the observed data. In this case, the *p*-value in the Durbin–Watson statistic test is less than 5.0%, and there is a sign of possible serial correlation at the 5.0% significance level. However, there is no distinguished pattern in the residual vs. run order plot.

**Figure 1:**
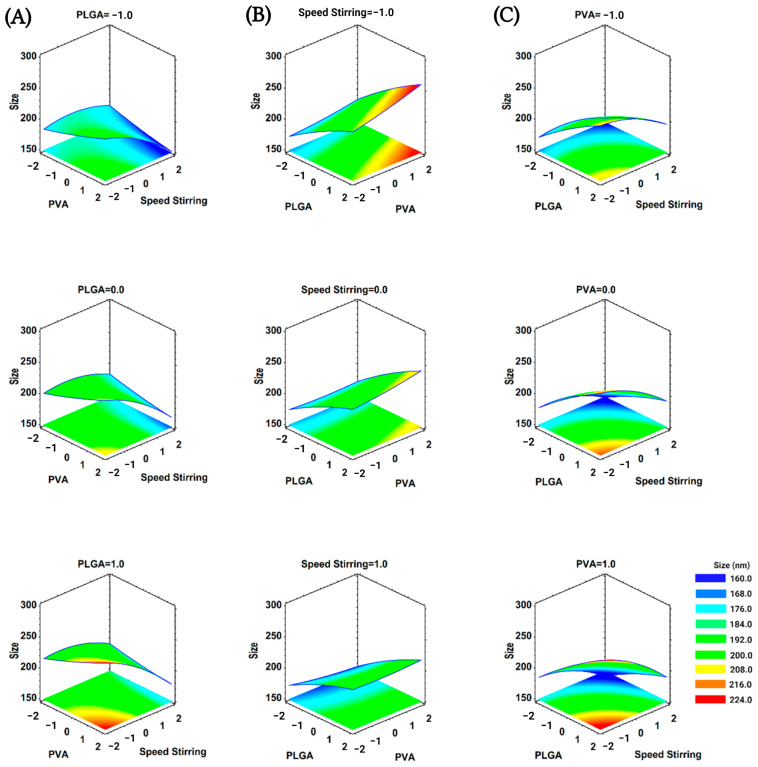
Graphics of estimated response for BNPs hydrodynamic size. Effect of (**A**) PLGA amount (−1 = 10 mg, 0 = 13 mg, 1 = 16 mg), (**B**) stirring speed (−1 = 800 rpm, 0 = 1050 rpm, 1 = 1300 rpm), and (**C**) PVA concentration (−1 = 1.0%, 0 = 2.0%, 1 = 3%).

**Figure 2:**
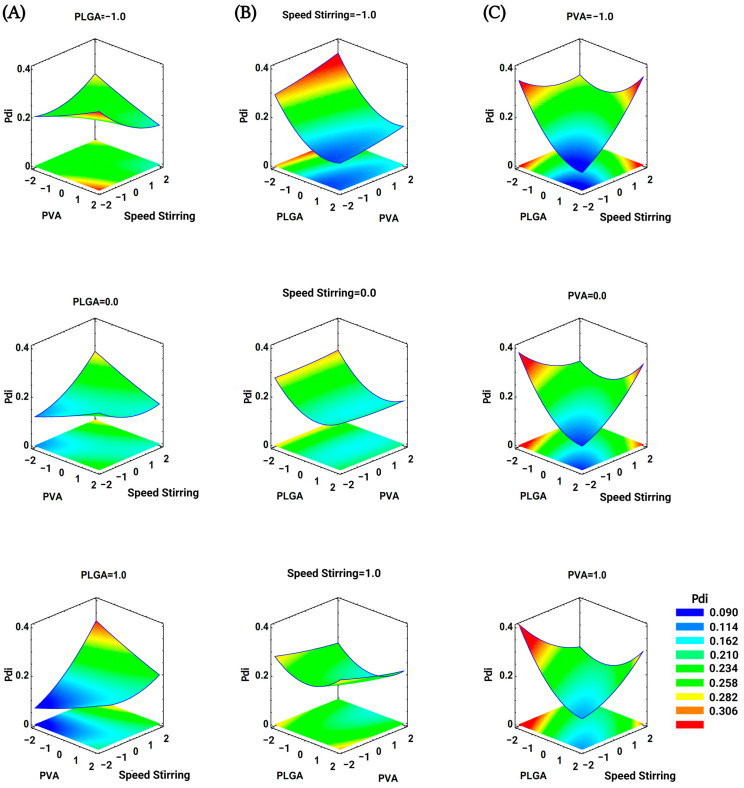
Graphics of estimated response for BNPs polydispersity index. Effect of (**A**) PLGA amount (−1 = 10 mg, 0 = 13 mg, 1 = 16 mg), (**B**) stirring speed (−1 = 800 rpm, 0 = 1050 rpm, 1 = 1300 rpm), and (**C**) PVA concentration (−1 = 1.0%, 0 = 2.0%, 1 = 3%).

We plotted the RSM with the CCD and the fitted modeling equations (Figure 1 and Figure 2). The RSM plots help to visualize how the PLGA amount and the stirring speed directly influence the size of the nanoparticles (Figure 1). When the PLGA amount increased, the nanoparticle size augmented over 200 nm (nearly 250 nm in the maximum levels plotted). Contrariwise, when the stirring speed increased, the nanoparticles reached a size close to 150 nm (at the maximum level of speed of stirring). However, the ANOVA indicated that the concentration of PVA (×2) had no significant influence. The graphical RSM in the PVA levels pointed out how the nanoparticle’s obtention can be controlled under 200 nm when the nanoprecipitation procedure is set with an adequate stirring speed and PLGA amount.

Similarly, the RSM graphics for the Pdi nanoparticles were obtained (Figure 2). In this case, the influence of five variables, including the PLGA amount (×1), the speed of stirring (×3), and their interactions with each other, result in a Pdi values response that is less homogeneous than the particle size response. When the PLGA amount is used at lower levels, the Pdi can vary around 0.2 and reach 0.23. When the PLGA amount increases, Pdi values take values close to 0.09 but reach values around 0.3. Regarding the speed of stirring, Pdi reached values from 0.09 to 0.33 at low speeds. However, at the stirring speed of around 1000 rpm, the Pdi has a narrower value, approximately within the range of 0.18 to 0.25. Additionally, the nanoparticles reduce their particle size at the greatest rates of speed stirring tested, resulting too in a constant Pdi value less than 0.3, which indicates an appropriate polydispersity. In this scenario, the smaller nanoparticles allow for more interactions with the stabilizer, which may prevent the eventual aggregation of the nanoparticles to one another, and decreases the value of Pdi. This effect is more marked when the amount of PLGA employed is at lower levels.

Even with a broad range of Pdi values, the optimization findings help choose the correct PLGA quantity, stirring speed, and PVA concentration to produce batches of nanoparticles that vary from mild (Pdi = 0.2) to arrow (Pdi = 0.1) dispersity. Likewise, nanoparticles smaller than 200 nm in size can evade fast clearance systems and prolong cell contact, allowing them to internalize and pass through biological barriers through passive diffusion and endocytosis [35]. Therefore, the process of nanoparticle optimization guarantees that the particle size and Pdi are ideal for the best possible interaction and cell internalization.

### 3.2. Entrapment Efficiency

**Figure 3:**
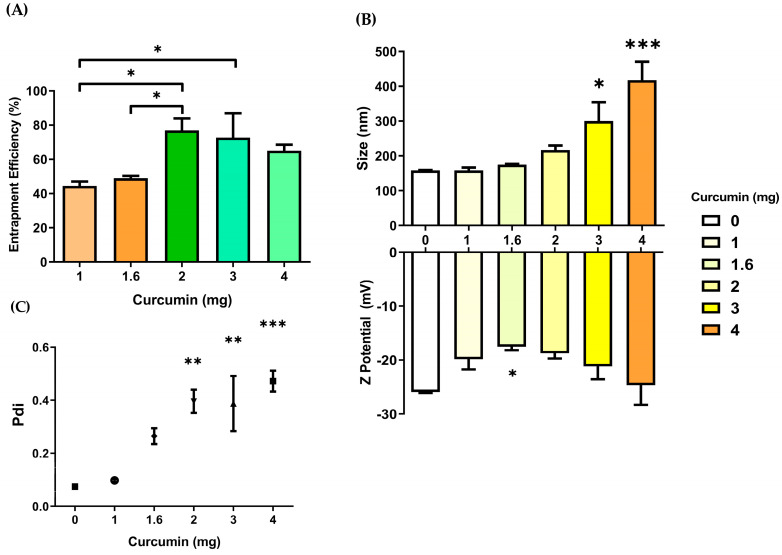
Curcumin entrapment efficiency and its effect on nanoparticle size, polydispersity index (Pdi), and zeta potential. (**A**) Entrapment efficiency analysis (* *p* < 0.05, ns = no significant, one-way ANOVA, Bonferroni’s multiple comparison test, *n* = 3). Effect of curcumin entrapment on (**B**) Size, zeta potential, and (**C**) Pdi (* *p* < 0.05, ** *p* < 0.01, *** *p* < 0.001, one-way ANOVA, Dunnett’s multiple comparison test, *n* = 3).

The preceding optimization allowed us to maintain desirable properties in the core of our nanoparticle. To assess the optimal curcumin entrapment, 16 mg of PLGA, with a stirring speed at 1300 rpm, and 3% *w/v* of PVA, were established as fixed factors. The ratios between PLGA and curcumin were assessed in ranges below, inside, and above, previously investigated by other researchers [7,36,37,38,39,40,41]. We examined the ratio of PLGA:curcumin (*w*:*w*) at 4:1, 5.3:1, 8:1, 10:1, and 16:1 to obtain the highest amount of entrapped curcumin. The ability of the nanoparticle system to load curcumin produced some interesting data (Figure 3). The amount of loaded curcumin increased to almost 70% when we added it in high amounts (about 3 or 4 mg) during testing. Despite this, when curcumin amounts were below 2 mg, the %EE narrowly reached 50%. With those %EE values, we examined the measurements of particle size, Pdi, and the stability of the nanoparticle through the zeta potential determination. When the ratio of PLGA:curcumin is closer, nanoparticle size and Pdi reach values around 500 nm and 0.4, respectively. When the ratio of polymer:drug is broad, the CNPs size (<200 nm) and Pdi (≈0.1) are narrower, without significant difference in BNPs. CNPs exhibited zeta potential values comparable to BNPs; only the CNPs batches produced with 1.6 mg of curcumin have a less negative value (over −20 mV). In those batches where the ratio of PLGA:curcumin tested is 10:1, the nanoparticle size (174.9 ± 4.4 nm) and zeta potential (−17.5 ± 1.1 mV) are comparable with previous works [40,42]. Nevertheless, the Pdi value of such batches can exceed 0.2, while batches produced with 1 mg of curcumin maintain a Pdi of less than 0.2, a particle size of 158.4 ± 14.3 nm, and a zeta potential of −19.8 ± 3.3 mV. Given that uniformity in size is a necessity for establishing an appropriate nanoparticle–cell contact, the particles that exhibit a lower Pdi are preferred. Therefore, we decided to utilize 1 mg of curcumin to create the CNPs for the subsequent experiments.

### 3.3. PEG:Trehalose Coating of Nanoparticles

#### 3.3.1. Effect of PEG:Trehalose Coating in Blank Nanoparticles and Curcumin-Loaded Nanoparticles

**Figure 4:**
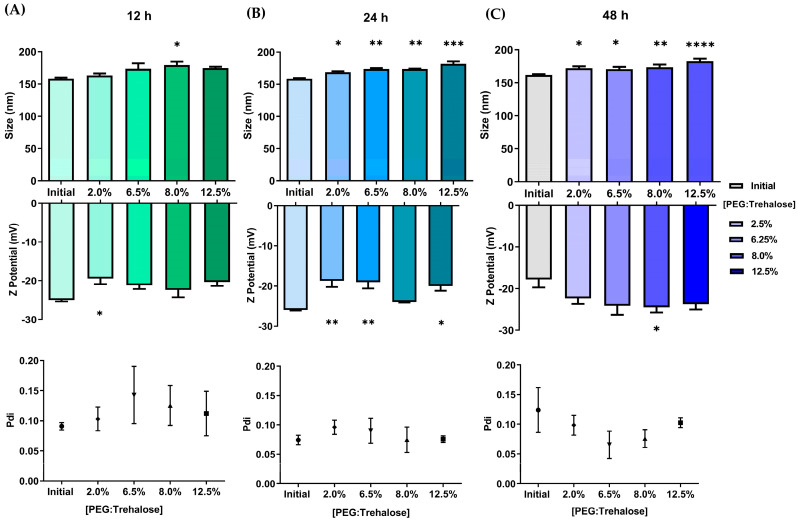
Coating effect on size, Pdi, and zeta potential of BNPs. BNPs were coated with PEG:Trehalose (1:1) for (**A**) 12, (**B**) 24, and (**C**) 48 h (* *p* < 0.05, ** *p* < 0.01, *** *p* < 0.001, **** *p* < 0.0001, one-way ANOVA, Dunnett’s multiple comparison test, *n* = 3).

During the BNPs coating process, the size and electrostatic charge of the nanoparticles tends to increase as the amounts of PEG:Trehalose and incubation time increase (Figure 4). The size of the nanoparticles ranged from 160 to 200 nm in experiments lasting 24 and 48 h. During the first 24 h of incubation, the zeta potential fluctuated between −20 and −30 mV. The coating process changed the particle interaction and increased the particle size, correlating with slower mobility and higher light diffraction in DLS. The electric charge increased due to PLGA, trehalose, and PEG interactions. Since trehalose molecules stabilize biological molecules through solvation and hydrogen bonding [43,44,45], trehalose may have reacted similarly with PLGA molecules in this environment, altering the interaction of the nanoparticles with the surrounding molecules.

**Figure 5:**
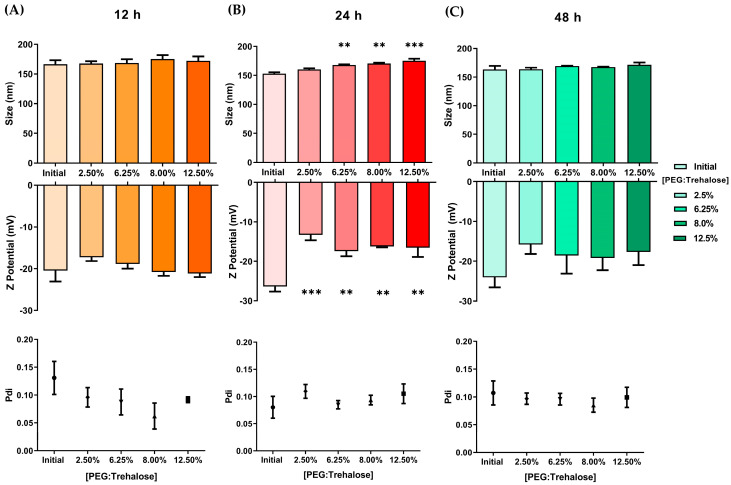
Coating effect on size, Pdi, and zeta potential of CNPs. CNPs were coated with PEG:Trehalose (1:1) for (**A**) 12, (**B**) 24, and (**C**) 48 h (** *p* < 0.01, *** *p* < 0.001, one-way ANOVA, Dunnett’s multiple comparison test, *n* = 3).

Figure 5 depicts size, Pdi, and zeta potential measurements during the CNPs coating process. In these experiments, nanoparticles increased significantly in size after 24 h of coating. Surprisingly, the particle sizes at 12 and 48 h were no different from the initial time. Curcumin loaded in nanoparticles appears to interfere with the probable interactions of nanoparticle surfaces with PEG and trehalose. The zeta potential exhibited a comparable impact, with only the incubation duration of 24 h showing significant differences. In this respect, the interaction of the nanoparticles with the coating materials may be altered over time. Interestingly, the size is maintained below 200 nm after the coating. These first alterations in size, Pdi, and zeta potential indicated the interaction of PEG:Trehalose with the nanoparticles and an effective coating in BNPs and CNPs. Based on these findings, we used 8% PEG:Trehalose and 24 h to obtain coated nanoparticles in all subsequent experiments. We chose these conditions based on the PLGA nanoparticle size (150 to 200 nm) and zeta potential (−13.2 to −19.3 mV) ranges that had shown decreased cytotoxicity in prior research [46].

**Figure 6:**
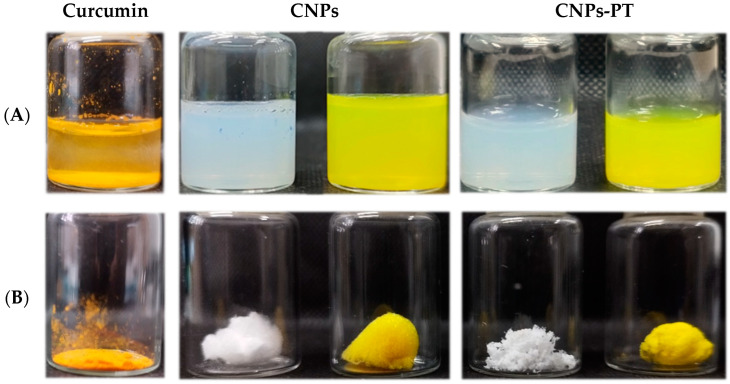
The physical appearance of (**A**) Water dispersion of curcumin powder, BNPs, CNPs, BNPs-PT, and CNPs-PT; (**B**) Curcumin powder and lyophilized of BNPs, CNPs, BNPs-PT, and CNPs-PT.

Figure 6 shows photographs of the curcumin’s appearance before and after preparing the nanoparticles. The improved curcumin–water interaction in the nanoparticulate system is noticeable. This formulation, which has no surface changes, may be investigated in various disease models and as a molecular detection tool [36,40]. In aqueous dispersions, there are no visible differences between nanoparticles and coated nanoparticles. The complete homogeneity of CNPs-PT and BNPs-PT indicates that the nanoparticles are stable following coating treatment. Figure 6 also shows pictures of lyophilized batches, and we found significant visual differences. Curcumin raw powder is denser and more compact than lyophilized CNPs. The BNPs powder is lighter and creates a cotton complex. On the other hand, CNPs also have a cotton appearance but are more crystalized and reflect light. Including the PEG:Trehalose coating procedure in BNPs and CNPs increases the powder density in both nanoparticle batches, whereas the light-reflecting feature in CNPs diminishes. Our findings show that the coating process caused observable physical changes in the batches of nanoparticles. The chemical composition, as measured by FTIR, stability, and temperature behavior, is explained in the following sections.

#### 3.3.2. Fourier Transform Infrared Spectra

**Figure 7:**
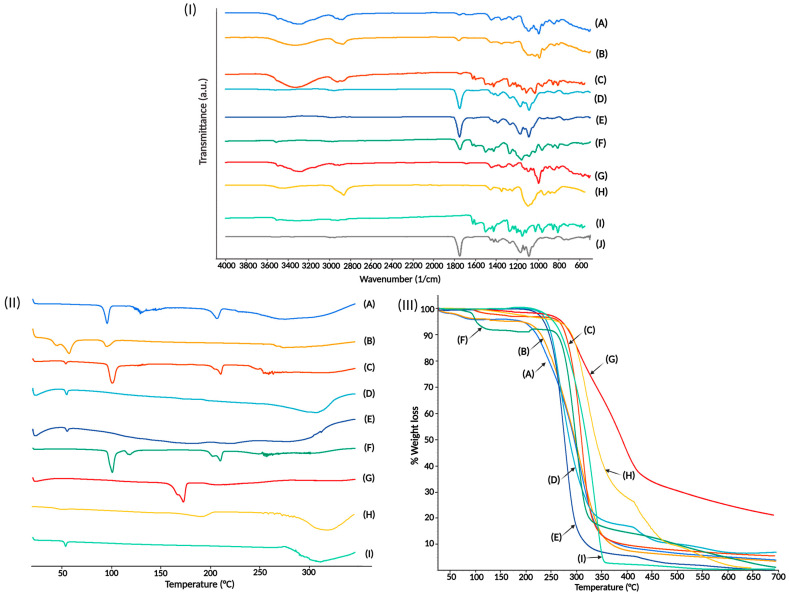
(**I**) FTIR Spectra of (A) CNPs-PT, (B) BNPs-PT, (C) Physical mixture (PLGA, curcumin, trehalose, and PEG), (D) CNPs, (E) BNPs, (F) Physical mixture (PLGA and curcumin), (G) Trehalose, (H) PEG, (I) Curcumin, and (J) PLGA. (**II**) DSC thermograms and (**III**) TGA of (A) CNPs-PT, (B) BNPs-PT, (C) Physical mixture (PLGA, curcumin, trehalose, and PEG), (D) CNPs, (E) BNPs, (F) Trehalose, (G) Curcumin, (H) PVA, and (I) PLGA.

The FTIR spectrum for PLGA nanoparticles is shown in Figure 7 (Section I). Typical signs of the carbonyl groups, stretching bands 1753 cm^−1^ from the two monomers in PLGA, are present. Stretching bands between 1300 and 1150 cm^−1^ corresponding to ester groups in PLGA may also be found [47]. The similarity between PLGA and BNPs spectra indicates that PLGA has not undergone any chemical modification during the nanoprecipitation process.

We obtained the FTIR spectrum for the physical mixture of PLGA and curcumin. Curcumin molecules exhibit stretching vibrations at 1628 cm^−1^, attributed predominantly to the overlapping stretching vibrations of alkenes (C=C) and carbonyl (C=O) characteristics, and C=C aromatic stretching vibration at 1427 cm^−1^ and ν(C-O) phenolic band at 1272 cm^−1^ [48,49]. The absorption band from the PLGA molecules was preserved at 1753 cm^−1^. The presence of the absorption bands for the molecules of PLGA and curcumin indicates that there is no in situ chemical interaction between them. The physical mixes showed noticeably pronounced absorption bands from both chemicals. However, the typical bands for curcumin were not seen in the FTIR spectra for CNPs. Likewise, most PLGA absorption bands do not indicate newly created covalent bonds. Although these phenomena could suggest the absence of curcumin in the batch, its yellow color indicates the presence of the compound in the nanoparticles (Figure 6). A possible explanation is that curcumin and the PLGA molecules around it help to create hydrogen connections between their C=O atoms. Other PLGA nanoparticles showed similar effects [42,50,51]; absorption curcumin bands were covered partially or totally by the PLGA nanoparticles complex.

We performed an FTIR study in raw materials, BNPs, CNPs, BNPs-PT, and CNPs-PT to assess the stable connection between the particles and the coating treatment. The peak signals for PLGA and curcumin were masked by the distinctive bands for trehalose and PEG in FTIR spectra for physical mixtures of raw materials. PLGA’s distinctive band at 1750 cm^−1^ was observed in FTIR spectra for BNPs-PT and CNPs-PT. Signals for the stretching bridge C-O-C, the main trehalose, and two crystal water molecules are visible at about 3500, 1000, and 954 cm^−1^, respectively [52]. Peaks at 2860 cm^−1^ for -CH_3_ stretching and C-O-C symmetrical stretching bands at 1100 cm^−1^ belong to PEG [53,54,55]. These measurements’ results indicate modifications to the molecular vibrational dynamics of the nanoparticles. The variations prove that PEG and trehalose constitute a molecular combination in the nanoparticles. A secondary chemical bond’s potential presence is supported by changes in absorption energy but not by newly formed covalent bonds [51].

#### 3.3.3. Differential Scanning Calorimetry

We show the DSC thermograms of our nanoparticles and raw materials in Figure 7 (Section II). Polymeric materials such as PLGA and PEG have a melting point below 100 °C. PVA shows a similar thermal behavior, with a thermal event at around 70 degrees and another at about 200 °C [56]. These polymers’ amorphous natures exhibit a modest signal for their thermal events and stability up to around 200 °C. On the other hand, crystalline compounds such as curcumin and trehalose have a stronger endothermic signal than polymers. The curcumin molecule has an endothermal event at 170 °C [57], whereas trehalose has two thermal events, a sharp one at 100 °C and a wider one at around 200 °C [58].

The DSC scan of our optimized BNPs showed an endothermic peak at 50 °C, potentially associated with the PLGA glass transition temperature [40], followed by thermal stability until over 250 °C. Thermograms for CNPs showed a similar thermal response to that of BNPs; similarly to other studies, we did not find the missing peak for crystalline curcumin at 173 °C [40,59]. This finding indicates the potential prevention of curcumin recrystallization during nanoparticle synthesis. Curcumin may be present in the nanoparticles in an amorphous state, a disordered crystalline phase, or a solid solution state, similar to docetaxel when synthesized in PLGA nanoparticles [60].

The DSC thermograms of BNPs-PT and CNPs-PT showed distinct thermal behavior. BNPs-PT displayed three thermal events related to interactions between the polymers PVA and PLGA at 45.5, 56.6, and 94.89 °C. The first peak exhibited two signals at 45.5 and 56.6 °C, close to the typical endothermic peaks of PLGA and PVA’s glass transition [61]. The third peak at 94.89 °C may be related to trehalose’s crystalline nature. Thus, the interactions between PLGA, PVA, PEG, and trehalose probably caused the changes in the temperatures at which endothermic signals were observed. The CNPs-PT exhibited three endothermal peaks at 95, 129.07, and 206.9 °C. These occurrences are mainly related to trehalose thermal characteristics [58]. However, the highest signal (at 129.7 °C) might be attributed to a trehalose alpha configuration [43]. The thermal stability of the nanoparticles was possibly a result of intermolecular interactions between all of its constituent molecules. Trehalose and PEG appear to provide the system with a completely different level of thermal stability, helping stabilize processes, including the warming of nanoparticles. Therefore, nanoparticles’ coating might significantly influence keeping their qualities until they reach the target cells.

#### 3.3.4. Thermogravimetric Analysis

We used TGA to evaluate the stability and thermal performance of nanoparticles and coated nanoparticles (Figure 7, Section III). The thermograms show consistent thermal degradation in both pure materials and nanoparticles. However, the nanoparticle systems had quicker degradation patterns than the materials did, due to a higher contact surface. The increased contact surface is associated with improved heat transfer [62,63]. Nevertheless, the rates of weight loss differed amongst the nanoparticle systems. BNPs showed a quicker thermal decomposition compared to the physical combination and the pure components. Stock PLGA was stable up to 200 °C, but decomposition began at 221 °C and continued over 350 °C, losing 95% of its weight [36,50]. However, BNPs lose 90% of their weight at 350 °C, perhaps due to the presence of PVA as a stabilizer. Stock PVA had a typical thermal degradation pattern consistent with earlier reports and degraded more slowly than PLGA [64]. Polyesters such as PLGA display a nonradical, backbiting ester exchange reaction involving the OH chain ends as a mechanism for deterioration at low temperatures. When the temperature rises, a radical chain scission process emits carbon monoxide, methylketene, ketene, and formaldehyde [65]. The CNPs had different thermal stability characteristics to BNPs, showing a slower weight reduction. Curcumin showed a weight loss at 100 °C, and degradation started at 200 °C; the first 5% of weight loss could be attributed to the presence of moisture. The crystalline structure of curcumin makes it more stable until 400 °C, where curcumin had a weight loss of 75% [66]. Due to the curcumin’s crystalline characteristics, the CNPs structure is more thermally stable than BNPs, which also results in a second stage of weight loss at around 412 °C. Thus, whereas CNPs had lost 80% of their weight, BNPs had lost 95% of their original weight.

BNPs-PT and CNPs-PT have extremely comparable thermal properties. Both exhibit little weight loss of 5% from 30 °C to 100 °C and thermal stability until 200 °C when both nanoparticles begin a rapid degradation and slow down around 350 °C with a 75% of weight loss. The early weight loss step can be attributed to moisture and trehalose coating, which is related to trehalose dehydration [36,62]. The trajectories of both samples in the thermogram intersect at about 350 °C in the second stage when weight loss is faster, and both samples lose 60% of the weight. At this temperature, the CNPs-PT had a quicker weight reduction than BNPs-PT. The first derivative of the TGA (DTA) was obtained to confirm if this event happened or whether there were disparities between the thermal patterns. The DTA revealed a peak for BNPs-PT at 308 °C and CNPs-PT at 305 °C. The heat distribution in the sample may change if curcumin is included in the nanoparticle system as other drugs [67]. In summary, a successful PEG:Trehalose coating was demonstrated through the distinct thermal behavior of coated nanoparticles, which TGA supported. Changes in thermal stability revealed the presence of molecules with a new thermal stability profile.

### 3.4. In Vitro Curcumin Release

**Figure 8:**
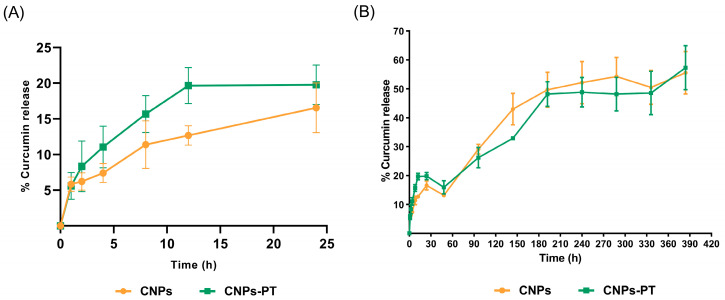
Curcumin release from CNPs and CNPs-PT. (**A**) Curcumin release from CNPs and CNP-PTs in the first 24 h monitored, CNPs exhibited slighter curcumin release than CNP-PTs. (**B**) Curcumin release for all the monitored time.

Over two weeks, we performed the in vitro curcumin release profiles for CNPs and CNPs-PT. Both release curves displayed a similar shape, and the classic burst effect was not substantial for the first 60 min (Figure 8). In the first 24 h, 20% of curcumin was released by both systems. It is feasible that a low amount of curcumin surrounding the nanoparticles and a more effective entrapment of curcumin are both responsible for the observed modest burst impact. The rate of release slows down after the first 24 h and reaches around 60% of release after 360 h. Drug dispersion via pores and a gradual release coupled with polymer breakdown might cause this second stage [68]. Moreover, both samples reached almost the same percentage of drug release in the last h of the experiment. Nevertheless, DE and MDT indicated a significant variation in the release of nanoparticles. CNPs had an MDT of 54.9 h and a DE% of 18.29%. CNPs-PT had an MDT of 72.87 h and a DE% of 17.89%. These values suggest that both systems could release curcumin over an extended period with a similar DE% [69,70]. Since CNPs and CNPs-PT differ by around 20 h on MDT, this could affect how much curcumin or other drug is released simultaneously. In other words, coated nanoparticles may be able to retain drugs for longer and release them 20 h later.

We fitted the release curves in the more typical mathematical models to characterize the release profile of nanoparticles. To define the way by which the release of curcumin is controlled, we adjusted various drug kinetic release models (See Table 5). The coefficient of determination (r^2^), Akaike information criterion (AIC), and model selection criteria (MSC) served as the factors to identify the optimal model [29,37,71]. Following these criteria, the Korsmeyer-Peppas is the best model to describe the release behavior of CNPs and CNPs-PT. The Korsmeyer-Peppas is a semi-empirical model that links the exponential nature of release with time. This concept proposes that curcumin follows the Power Law and is released from a polymeric matrix, indicating that drug transport, relaxation, and diffusion likely occur throughout both systems’ dissolution processes. Drug release behavior is related to the exponent “n” (exponent of release) in the Korsmeyer-Peppas equation F = kKPt^n^. The release might adhere to or deviate from Fick’s law, depending on the “n” number [72,73]. Our systems of nanoparticles scored an “n” value of 0.49 for CNPs and 0.45 for CNPs-PT. These “n” values categorize both systems as spherical matrix forms associated with polymeric matrix phenomena. The rate of diffusion governs dissolution in this system more than the rate of polymeric chain relaxation [74]. By analyzing these data, we could determine that we had successfully created nanoparticles that could release their active component over an extended period, comparable to previous PLGA-based systems [68,75]. The active ingredient in the model is released identically despite the surface alteration, which does not affect the stability of the nanoparticle core.

### 3.5. In Vitro Cytotoxicity Assay

**Figure 9:**
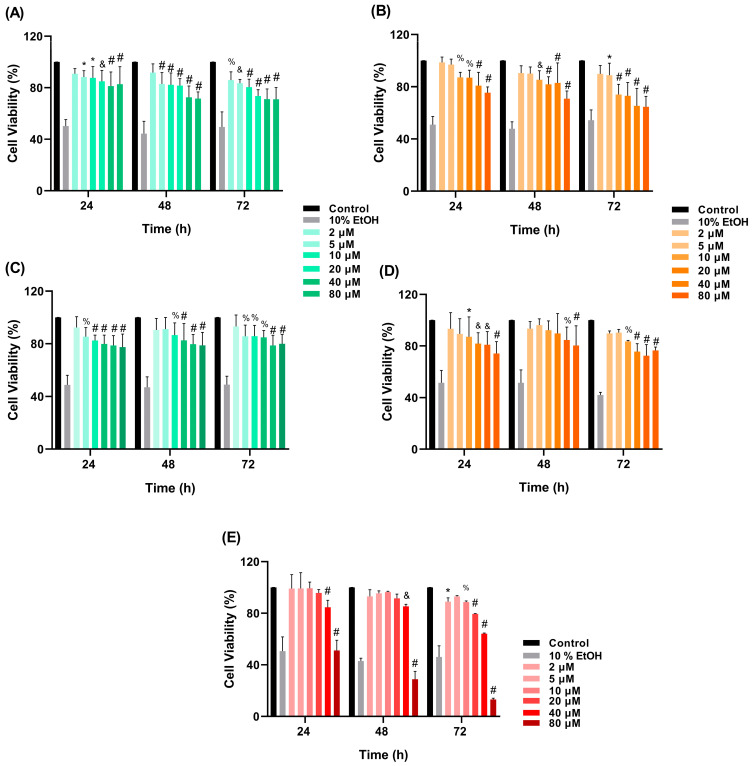
Nanoparticles In vitro cytotoxicity assay in SH-SY5Y cells. Treatments: (**A**) BNPs, (**B**) CNPs, (**C**) BNPs-PT, (**D**) CNPs-PT, (**E**) Curcumin (# *p* < 0.0001, & *p* < 0.001, % *p* < 0.01, * *p* < 0.1 vs. Control, two-way ANOVA, Dunnett’s multiple com-parison test, *n* = 3).

For cytotoxicity assays, SH-SY5Y cells were exposed to curcumin, BNPs, BNPs-PT, CNPs, and CNPs-PT for 24, 48, and 72 h, respectively. In all treatments, cells decreased their metabolic activity in a concentration- and time-dependent way (Figure 9). Treatments with BNPs and CNPs (20–80 µM) for 48–72 h reduced SH-SY5Y cells’ survival below 90%. BNPs-PT 5–80 µM decreased the cells’ survival level to below 90% at 24–72 h, whereas BNPs-PT 2 µM maintained cell survival levels at 90% at 72 h. Administration of CNPs-PT (10–80 µM) for 24 and 72 h decreased cell survival below 90%. However, cells treated with CNPs-PT 2–5 µM maintained cell survival values between 90 and 100% throughout all 72 h. At all time frames studied, curcumin 2–10 µM maintained cell survival between 90 and 100%. Previous studies demonstrated that curcumin in concentrations between 5–100 µM significantly decreases SH-SY5Y cells’ metabolic activity [34]. Moreover, curcumin concentrations of 5 µM had activity in SH-SY5Y neural disease models [76,77]. Therefore, curcumin concentrations below 5 µM (such as our CNPs or CNPs-PT) can be effective in cell culture models without compromising cell viability. Interestingly, the survival of SH-SY5Y cells was maintained above 60% in the treatments with CNPs and CNPs-PT 20, 40, and 80 µM at the three times examined. On the other hand, the survival of SH-SY5Y cells considerably declined after 24 h of exposure to curcumin at dosages of 20, 40, and 80 µM, and the surviving cells continued to reduce over the subsequent h. The information above shows that CNPs and CNPs-PT significantly diminish the toxicity of curcumin. In this regard, a desirable attribute (and practical aspect of polymeric nanoparticles promoted with a prolonged release period) is reducing drugs’ cytotoxic effect [78,79].

### 3.6. Nanoparticles Cell Uptake

**Figure 10:**
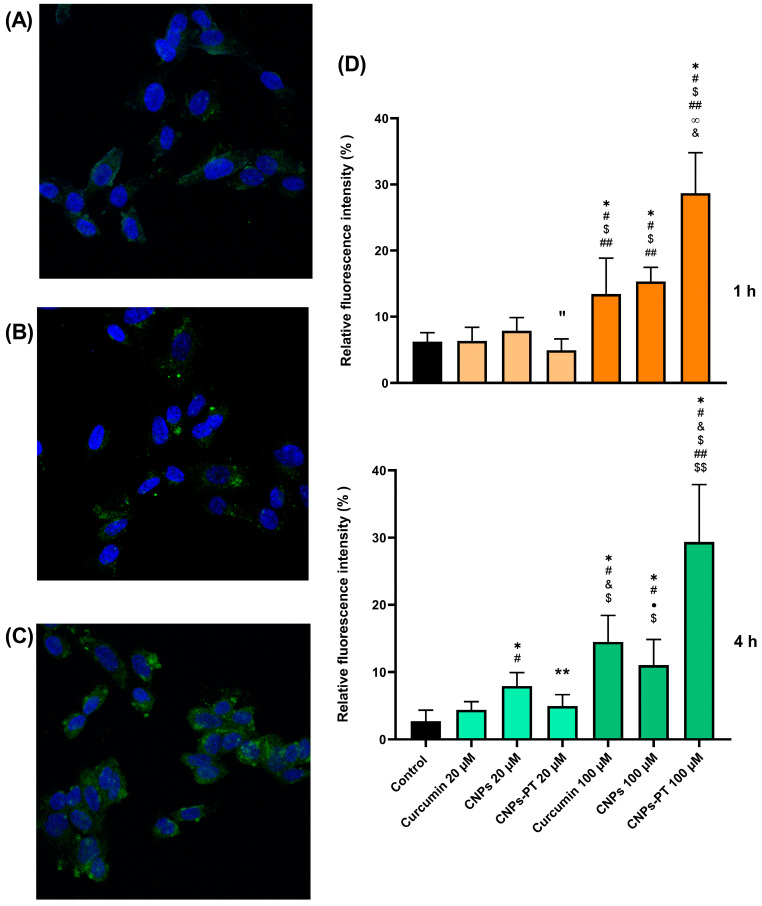
Confocal analysis of curcumin autofluorescence and DAPI staining in SH-SY5Y cells. Micrographs of SH-SY5Y exposed to (**A**) Curcumin 100 µM, (**B**) CNPs 100 µM, and (**C**) CNPs-PT 100 µM for 4 h. (**D**) Curcumin fluorescence intensity inside cells for exposure of nanoparticles. Treatments: control (black), curcumin, CNPs and CNPs-PT 20 µM (light orange), curcumin, CNPs and CNPs-PT 100 µM8 (dark orange) for 1 h (top; one-way ANOVA Tukey’s multiple comparisons, * *p* < 0.0001 vs. Control, # *p* < 0.0001 vs. curcumin 20 µM, $ *p* < 0.0001 vs. CNPs 20 µM, “ *p* = 0.004 vs. CNPs 20 µM, ** *p* < 0.0001 vs. CNPs-PT 20 µM, ∞ *p* < 0.0001 vs. Curcumin 100 µM, & *p* < 0.0001 vs. CNPs 100 µM) and, control (black), curcumin, CNPs and CNPs-PT 20 µM (light green), curcumin CNPs and CNPs-PT 100 µM (dark green) for 4 h (bottom, one-way ANOVA Tukey’s multiple comparisons; * *p* < 0.0001 vs. Control, # *p* < 0.0001 vs. Curcumin 20 µM, ** *p* = 0.0018 vs. CNPs 20 µM, ● *p* = 0.0154 vs. CNPs 20 µM, & *p* < 0.0001 vs. CNPs 20 µM, $ *p* < 0.0001 vs. CNPs-PT 20 µM, ## *p* < 0.0001 vs. Curcumin 100 µM, $$ *p* < 0.0001 vs. CNPs 100 µM).

In independent experiments, SH-SY5Y cells were incubated with 20 µM and 100 µM of curcumin and corresponding amounts of CNPs and CNPs-PT for 1 and 4 h (Figure 10). As a control, we also assessed BNPs and BNPs-PT. Using curcumin’s photoreactivity, we monitored the internalization of the nanoparticles into the cells. In previous studies, curcumin (1 µM), curcumin-loaded nanoparticles (1 µM), and time intervals between 1 and 24 h were used to examine the cell internalization [80,81,82,83]. Nevertheless, nanoparticle internalization to cells may occur in a few minutes and can be monitored in real-time using curcumin as a probe [83]. Additional factors such as nanoparticle concentration, curcumin quantity, nanoparticle-cell contact duration, and cellular type might influence nanoparticle absorption [10,80].

Confocal analyses revealed a weak fluorescence intensity of curcumin, CNPs, and CNPs-PT (curcumin 20 µM in all cases) at 1 and 4 h within SH-SY5Y cells. However, we could not find the difference between the treatments, and the signal intensity was equivalent to the controls without curcumin. In this respect, curcumin exhibits significant fading upon photo-illumination and disintegration in pH ≥ 7 environments [4]. Thus, the confocal laser most likely caused curcumin breakdown and excitability loss at this concentration. On the other hand, we found substantial fluorescence intensity signals inside cells treated with curcumin, CNPs, and CNPs-PT (curcumin 100 µM in all cases) at both periods (1 and 4 h). We observed the maximum fluorescence in captures of cells treated with CNPs-PT using eye visualization. To confirm that the signal was more robust in particular cells, we used Image J software to qualitatively analyze the signal intensity by separating channels and measuring the signals in the green channel layer. We measured the signal intensity of curcumin within cells (minus background) on a cell-by-cell basis using micrographs that revealed cells with a favorable appearance from three wells. After 1 h of exposure, the relative percentages of fluorescence for curcumin, CNPs, and CNPs-PT (curcumin 100 µM in all cases) were 11.91 ± 3.36, 16.09 ± 3.792, and 25.52 ± 7.58, respectively. Furthermore, curcumin, CNPs, and CNPs-PT readings for cell images incubated for 4 h were 14.50 ± 3.93, 11.40 ± 3.60, and 30.49 ± 7.57, respectively. In 1 and 4 h, measurements showed that cells treated with CNPs-PT had higher fluorescent signals inside.

Therefore, the data from this set of experiments support our hypothesis that PEG:Trehalose coating treatment enhances cell uptake of nanoparticles. PEG incorporation may cause cellular membranes to be damaged and increase the uptake of nanoparticles [17], whereas trehalose can help to stabilize molecules by encircling molecular complexes such as protein [43,44]. Likewise, our findings are consistent with the idea that trehalose might improve the absorption of nanoparticles by creating a more suitable environment [25]. Trehalose can internalize cells through fluid endocytosis by producing vesicles made from plasmatic membranes in concentration-dependent ways [84]. According to some reports, trehalose inhibits the function of the SLCA2A hexose transporter family and indirectly controls the autophagy process [85,86,87]. In summary, the modification of the surface of CNPs-PT by PEG and trehalose can lead to their internalization via cell membrane rupture, membrane fluidization, and enhanced interaction of nanoparticles with hexose transporters.

## 4. Conclusions

In this work, we synthesized PEG:Trehalose-coated nanoparticles that can carry chemically complicated substances with properties such as very low hydrophilicity, including curcumin. The in vitro drug release test revealed that even after at least 72 h of stability, some of the curcumin would not be released from the particles. Consequently, our nanosystem’s frequent dosing might reduce curcumin toxicity and boost bioavailability. The adsorption of PEG:Trehalose effectively increases cell absorption of nanoparticles. The enhanced cellular absorption of nanoparticles can result in a significant amount of curcumin inside the cells. The slow release of high doses of curcumin from nanoparticles inside neurons might improve the antioxidant and anti-inflammatory activity against brain diseases.

## Figures and Tables

**Table 1:** Codified values in Central Composite Design for Nanoprecipitation process for PLGA nanoparticles synthesis.

Codified Value	PLGA (mg) (*x*_1_)	PVA (%*w*/*v*) (*x*_2_)	Stirring (rpm) *(x*_3_*)*
−1.68 (−α)	7.95	0.32	630
−1	10.0	1.0	800
0	13.0	2.0	1050
1	16.0	3.0	1300
+1.68 (+α)	18.04	3.68	1470

**Table 2:** Independent variables and responses for central composite design in nanoprecipitation process for PLGA nanoparticles synthesis.

	Independent Variables			Response	
Run	PLGA (mg) *(x*_1_*)*	PVA (%*w*/*v*) *(x*_2_*)*	Stirring (rpm) *(x*_3_*)*	Size (nm) *(y*_1_*)*	Pdi *(y*_2_*)*
1	18.04	2	1050	227.78 ± 11.561	0.160 ± 0.019
2	10	3	1300	179.63 ± 12.903	0.222 ± 0.067
3	13	2	1050	213.38 ± 5.298	0.133 ± 0.033
4	7.95	2	1050	190.74 ± 12.377	0.249 ± 0.042
5	10	1	1300	174.33 ± 2.972	0.201 ± 0.024
6	13	2	1050	208.83 ± 5.266	0.183 ± 0.020
7	13	2	629.5	226.18 ± 5.360	0.120 ± 0.027
8	13	2	1050	215.06 ± 6.057	0.155 ± 0.026
9	13	0.3	1050	210.29 ± 2.701	0.163 ± 0.013
10	13	2	1470.4	184.56 ± 5.593	0.240 ± 0.023
11	16	1	1300	205.76 ± 2.225	0.196 ± 0.009
12	13	3.6	1050	216.63 ± 6.060	0.157 ± 0.028
13	10	1	800	186.61 ± 2.601	0.222 ± 0.074
14	16	1	800	217.96 ± 3.743	0.139 ± 0.007
15	13	2	1050	197.02 ± 2.605	0.164 ± 0.013
16	16	3	800	235.18 ± 5.824	0.159 ± 0.019
17	13	2	1050	194.69 ± 2.152	0.166 ± 0.011
18	13	2	1050	203.71 ± 1.438	0.163 ±0.013
19	10	3	800	185.62 ± 5.867	0.208 ± 0.043
20	16	3	1300	201.37 ± 6.279	0.174 ± 0.018

**Table 3:** Analysis of variance for nanoparticles size from central composite design for nanoprecipitation technique optimization for PLGA nanoparticles synthesis.

Source	Sum of Squares	d.f.	Mean Square	F-Ratio	*p*-Value
*x*_1_: PLGA	8468.26	1	8468.26	95.58	0.0000
*x*_2_: PVA	169.88	1	169.88	1.92	0.1864
*x*_3_: SpeedStirring	4806.13	1	4806.13	54.25	0.0000
*x*_1_ *x*_1_	46.34	1	46.34	0.52	0.4807
*x*_1_ *x*_2_	27.22	1	27.22	0.31	0.5875
*x*_1_ *x*_3_	74.55	1	74.55	0.84	0.3735
*x*_2_ *x*_2_	8.72	1	8.72	0.10	0.7581
*x*_2_ *x*_3_	314.65	1	314.65	3.55	0.0790
*x*_3_ *x*_3_	251.48	1	251.48	2.84	0.1127
Blocks	174.11	2	87.05	0.98	0.3972
Lack of fit	3154.95	33	95.60	1.08	0.4543
Pure error	1328.95	15	88.60		
Total	18,823.30	59			

Two effects have *p*-values less than 0.05 (marked in bold), r2 = 76.179 percent, r2 (adjusted for d.f.) = 70.72 percent Standard Error of Est. = 9.41259, Mean absolute error = 6.8946, Durbin–Watson statistic = 2.3644 (*p* = 0.8731).

**Table 4:** Analysis of variance for Pdi from central composite design for nanoprecipitation technique optimization for PLGA nanoparticles synthesis.

Source	Sum of Squares	d.f.	Mean Square	F-Ratio	*p*-Value
*x*_1_: PLGA	0.024360	1	0.024360	30.45	0.0001
*x*_2_: PVA	0.000003	1	0.000003	0.00	0.9468
*x*_3_: SpeedStirring	0.005517	1	0.005517	6.90	0.0191
*x*_1_ *x*_1_	0.012436	1	0.012436	15.55	0.0013
*x*_1_ *x*_2_	0.000032	1	0.000032	0.04	0.8426
*x*_1_ *x*_3_	0.012880	1	0.012880	16.10	0.0011
*x*_2_ *x*_2_	0.000078	1	0.000078	0.10	0.7588
*x*_2_ *x*_3_	0.005046	1	0.005046	6.31	0.0240
*x*_3_ *x*_3_	0.003148	1	0.003148	3.94	0.0659
Blocks	0.003446	2	0.001723	2.15	0.1506
Lack of fit	0.054255	33	0.001644	2.06	0.0693
Pure error	0.011999	15	0.000799		
Total	0.132069	59			

Five effects have *p*-values less than 0.05 (marked in bold), r^2^ = 49.8328 percent, r^2^ (adjusted for d.f.) = 38.3362 percent, Standard Error of Est. = 0.0282837, Mean absolute error = 0.0262004, Durbin–Watson statistic = 1.42977 (*p* = 0.0070).

**Table 5:** Release kinetic data modeling of CNPs and CNPs-PT. The first section of the table corresponds to the first stage of release, burst effect. The second section of the table to the gradual release.

Particles	Parameters	Zero Order	First Order	Higuchi	Korsmeyer–Peppas	Hixon–Crowell
		f = k × t	ln(1 − f) = −kt	f = kt^1/2^	F = k_KP_·t^n^	1 − 1(1 − f)^1/3^ = −k × t
CNPs	r^2^	0.756	0.833	0.953	0.967	0.812
	AIC	93.5	86.2	70.2	68.7	88.6
	MSC	1.40	1.92	3.06	3.17	1.75
CNPs-PT	r^2^	0.598	0.689	0.895	0.953	0.656
	AIC	96.4	93.6	79.3	71.2	94.39
	MSC	1.02	1.21	2.24	2.82	1.16
**Particles**	**Parameters**	**Makoid–Banakar**	**Peppas–Shalin**	**Baker–Lonsdale**		**Weibul** **l**
		f = kMB t^1/2^e(^−ct^)	f = k1 × t^1/2^+k2·t	3/2 ∗ [1 − (1 − F/100)^(2/3)] − F/100 = kBL ∗ t		f = Fmax·[1 − e^(−tˆβα)^]
CNPs	r^2^	0.969	0.969	0.952		0.968
	AIC	69.6	71.3	71.3		70.3
	MSC	3.11	3.10	2.9		3.05
CNPs-PT	r^2^	0.958	0.939	0.902		0.950
	AIC	71.9	75.0	78.3		73.9
	MSC	2.76	2.54	2.31		2.63

r^2^: coefficient of determination; AIC: Akaike information criterion; MSC: Model Selection Criterion.

## Data Availability

Not applicable.
